# Identifying the Incidence of Exercise Dependence Attitudes, Levels of Body Perception, and Preferences for Use of Fitness Technology Monitoring

**DOI:** 10.3390/ijerph15122614

**Published:** 2018-11-22

**Authors:** Dana Badau, Adela Badau

**Affiliations:** 1 Department of Human Movement Sciences, University of Medicine, Pharmacy, Sciences and Technology of Targu Mures, 540139 Targu Mures, Romania; 2 Department of Physical Education, University of Medicine, Pharmacy, Sciences and Technology of Targu Mures, 540139 Targu Mures, Romania; adela.badau@umftgm.ro

**Keywords:** health, physical exercise, technology with monitoring, exercise addiction

## Abstract

*Background*: The study was focused on identifying the relationship between the incidence of exercise dependence attitudes, the level of body perception, and preferences for use of fitness technology monitoring. *Methods:* We investigated 241 students from physical education and sports specializations. We used a questionnaire structured in 5 parts: personal information (age, gender, weight, height, and institutional membership), Exercise Dependence Scale-R (EDS-21), the Compulsive Exercise Test (CET), Body perception questionnaire, and Fitness Monitoring Technology. *Results*: Application of EDS-21 revealed 8.3% with dependence and CET with 13.4%. Body mass index (BMI) in combination with self-image body: 18 (7.4%) of subjects over-estimated themselves, 18 (7.4%) of subjects underestimated themselves, 38.6% of the subjects were satisfied with their appearance, 17% were very satisfied and none were dissatisfied. A total of 36.1% thought they had the correct weight and 8.7% thought of losing weight. The most preferred monitoring technologies were the treadmill and the smartphone’s fitness applications. A total of 11.6% of the subjects always used technologies, and 17% of subjects never did so. *Conclusions*: In order to identify the level of incidence of exercise dependence, body perception, and preference and frequency of use of monitoring technology, it is necessary to expand the level of knowledge about health and physical activities. The approach would diminish medical incidences of addiction, improve proactive behaviors, and correct prohealth.

## 1. Introduction

### 1.1. Theoretical Background and Novelty of the Study

The investigation was focused on the sustainable development of human beings by promoting positive, active and optimal behaviors and mentalities. Consequently, this will lead to the development of human abilities, promoting health and improving the quality of life. Practicing regular physical activity has effects both on mental and physical health; however, some individuals spend an excessive amount of time exercising, which can lead to the formation of physical impediments [1]. The scientific literature contains few studies which have approached exercise dependence, body perception and use of fitness technology monitoring in an interrelated manner. Students from physical education and sports are mostly active athletes with relevant information about the benefits of exercising, the negative effects of exercise, the technological means used in practice and the formation of proactive and prohealthy behaviors and knowledge about nutrition, physiology of effort, restoration, physical recovery, psychology, and behavioral sciences [2].

The novelty of our study consists in evaluating students from the physical education and sports profile, future physical specialists who acquire knowledge and skills during their academic training for promoting an active and healthy lifestyle and combating the risks and negative effects of excessive exercising. Despite specialized training, there is a certain level of incidence of exercise addiction among students in the physical education and sports program [3,4].

Several studies have shown that exercise dependence is based on an erroneous perception of body image. A new trend in physical activity is the continuous monitoring of training with the help of various fitness technologies, which may increase the incidence of exercise addiction.

Identifying the factors that determine exercise-dependent behaviors among students in physical education and sports can help optimize academic training by increasing the role of education and health counseling disciplines. Addiction to making specific physical efforts, which experts have called addiction exercises, is currently not included in the clinical use of diagnosis and assessment manuals [5]. Indeed, numerous studies have shown that this nosological change is associated with obsessive-compulsive disorder [6,7] or addictive processes [8,9,10,11]. Currently, therapeutic approaches to exercise dependency primarily target the use of cognitive-behavioral therapies and methods to lessen other behavioral dependencies [12,13]. These can lead to the desire to over-repeat a physical program for a long period of time, to self-indulge in physical performance, and to permanently improve body image and self-confidence.

Exercise-dependent attitudes can be caused by psychic and medical factors [4,14,15,16]. Exercise addiction can cause the increased incidence of fractures, sprains, luxation and other functional problems of the body [17,18,19,20,21,22]. From a social point of view, exercise addicts often isolate themselves for practice, lack reality perception, and minimize social relationships [8,23]. From an athletic point of view, exercise dependence can also cause a decrease in physical and sports performances due to overloads and physical exhaustion [24,25,26]. The three-dimensional approach of our study on exercise addiction, body perception, and the use of fitness-based monitoring technology will improve our understanding in proactive aspects of promoting exercise and the development of proactive and prohealth correct behaviors.

### 1.2. Exercise Dependence–Theoretical Approaches

Compulsive exercise has been defined as a rigid and highly driven urge to be physically active, in association with a perceived inability to stop exercising despite the individual’s awareness of possible negative consequences [27,28]. Most studies on exercise-related addiction have shown association with eating disorders or some psychological problems [29,30,31]. However, it can also produce changes in the daily routine due to poor health, and stressful social and professional conjunctions [32], with a great preponderance for fitness instructors and coaches [12,33,34]. Exercise addiction is influenced by age, gender, mood, and personality [35,36,37]. Exercise addiction includes o series of psychometric components systematized on the following subscales: tolerance, withdrawal, lack of control, intention effects, time, reduction in other activities, continuance [8,12,38]. Meyer C. et al. identified 5 scales, namely, avoidance and rule-driven behavior, weight control exercise; mood improvement; lack of exercise enjoyment, and exercise rigidity [29].

Analyses of all exercise dependency assessment subscales provide relevant information on the incidence of risk-free, risk-taking or dependent subjects. Regarding physical activities, Freimuth et al. proposed 4 stages of behavioral identification: (1) Recreational exercise, practiced in the leisure time in order to increase quality of life, (2) Risk exercise related to effective control, (3) Problematic exercise focused on organizing the daily program according to training, exceeding individual effort limits and (4) Sport dependence-involved a focus on physical training sessions and a reduction of social and professional integration [12]. In terms of exercise motivations, these are multiple, being both intrinsic and extrinsic. Studies show a relatively equal prevalence of exercise addiction among individual sports practitioners and team sports, but the motivations are different. For sports teams, these are pleasure and competition. Individual sports practitioners mainly targeted health and weight loss, and boys were more motivated than girls [39,40,41]. In this regard, we believe that the effects of exercise addiction can also be reflected in body perception, body image, weight appreciation, and the influence of weight optimization motivators, which we also wanted to take into account in this study.

### 1.3. Body Image Perception—Theoretical Approaches

According to researchers, the relationship between body image perception and exercise addiction is still insufficiently studied. Some studies have shown a positive influence between the two components [37,42,43], and others a negative influence between self- body imagine and exercise addiction [37,44]. Body image is a combination of the perception of one’s own physical aspect which is associated with emotional states and intellectual capabilities. As a rule, those who practice sports choose sports patterns that are confirmed by national or worldwide elites and promoted in the media, and body perception is often used in these models [45,46]. Theoretically, the body image for each individual coincides with a relative degree to the physical body of the person and are influenced by contexts specific to the physical and social environment [47,48]. Recent studies highlight that students perceived their body as being 50% over the normal weight. For physical exercise practitioners, this perception is much closer to reality, i.e., to their correct weight [49,50]. The influence of media on body image has negative effects on young students, promoting subjective and unhealthy practices at the expense of practicing sport and maintaining health [51]. Regarding the perception of body images among the young population, it was found that men tended to have a more positive perception than women [52,53].

### 1.4. Theoretical Approaches in Using Fitness Technologies with Monitoring

Practicing physical exercises to optimize health, improve motor capacity, and form a correct body image are influenced by a number of environmental and training factors, and by the technologies used. The use of technology with monitoring can help improving knowledge about the typology of fitness programs; monitoring the volume, intensity, and complexity of the effort during the training, obtaining and gathering accurate information in real time, giving the functional and physical parameters of the body during the effort, etc.

The technology used for health and fitness enhanced a global emphasis. It became the top worldwide fitness trend in 2016 and 2017 [54,55,56]. The development of fitness-specific technologies with monitoring is being continuously developed and improved to be used in the field of education, physical activity, sports medicine, and functional recovery [57,58,59,60,61]. Effort and motion assessment technologies are used to diagnose and improve the locomotor system and the major functions of the body (circulatory, respiratory) while optimizing specific physical activity programs such as physical education, physical recreation, or sports performance [62,63,64,65,66]. The use of these technologies is increasing because of their reliability, the facility of selecting pre-established training programs and evaluation, based on physiological responses in real time during the effort. In addition, fitness monitor technologies allow the collection of biometric information and basic training and physiological parameters both during rest and during exercise.

## 2. Experimental Section

### 2.1. Aims

Promoting proactive and pro-health behaviors through the use of various technologies are important components of education and health. In this respect the main goals of the study were:

- identifying the addictive attitudes regarding physical exercise among students from physical education and sports specializations,
- identifying the level of appreciation of body perception,
- identifying the preferences of using the fitness technologies with monitoring during training.

### 2.2. Research Design

We recruited 297 students from physical education and sports programs for our study. After reviewing the online questionnaires from the students, only 241 students were qualified for the research. The excluding criteria were that not all items of the questionnaires were filled in by the students and that they did not practice physical activity regularly during the last three months. The study was conducted in accordance with the ethics rules, with research standards from the Helsinki Declaration of 1964 and its amendments. All participants signed the inform consent forms (ethical no. UTB72/16 February 2018).

### 2.3. Participants

The study included 241 male students of the Physical Education and Sports academic program of which 134 (56.84%) students were studying a degree in it and 104 (43.16%) students were studying their masters. Distribution of the institutional membership of the study group: University of Medicine, Pharmacy, Sciences, and Technology of Targu Mures = 66 (27.38%) students, Transilvania University of Brasov = 89 (36.92%) students, and Dunarea de Jos University of Galati = 86 (35.86%) students. The average age of the group was 24.58 ± 4.08 years, with an average weight of 79.69 ± 14.37 kg and an average height of 176.80 ± 10.02 cm. All participants in this study were volunteers.

## 3. Results

The most relevant results and descriptive information were structured in 3 subsections corresponding to the three objectives of the study.

### 3.1. Study on Identifying the Incidence of Compulsive Attitudes and Exercise Addiction

Within these first subsections, we focused on the analysis of the two standardized questionnaires: the Exercise Dependence Scale-R (EDS-21) (Table 1.) and Compulsive Exercise Test (CET) (Table 2).

#### 3.1.1. The Exercise Dependence Scale-R (EDS-21)

The overall assessment of EDS-21 (Table 1) revealed that the recorded values were statistically significant for *p* < 0.05, thus 3.61 ± 0.46; t (df 20) 35.45. The results for each item of the questionnaire highlighted by the Z values of the Kolmogorov–Smirnov test were statistically significant for *p* < 0.05. For the whole questionnaire, the Keiser–Meyer–Olkin measurements verified the sampling adequacy for analysis, KMO = 0.766, which are above Keiser’s criteria (>0.5). Bartlett’s test of sphericity χ^2^ (210) = 3590.84, *p* < 0.0001, indicated that the correlation between these items was sufficiently large for PCA. The Cronbach’s Alpha coefficient for EDS-21 including 21 items was α = 0.909, suggesting that the questionnaire had a very high internal consistency. For subsections, the Cronbach’s Alpha coefficients were Withdrawal Effects α = 0.734, Continuance dependence α = 0.614, Tolerance α = 0.842, Lack of Control α = 0.739, Reduction in Other Activities α = 0.666, Time 0.831, Intention Effects α = 0.854, suggesting that all the subsections of the questionnaire had a high (over 0.600) and very high (over 0.800) internal consistency.

Per total EDS-21: 14.28% of subjects were awarded 6 points, 16.71% of subjects were awarded 5 points, 21.05% of subjects were awarded 4 points, 15.93% of subjects were awarded 3 points, 14.35% of subjects were awarded 2 points, and 17.68% of subjects were awarded 1 point. The EDS-21 questionnaire (Table 2.) recorded an average arithmetic value of X = 3.61 and an average value of the 871 ± 112.71 points. On the EDS-21 subscales, the recorded media values were as follows: Withdrawal Effects X = 3.16, X∑ = 749 points; Continuance dependence X = 3.44, X∑ = 831 points; Tolerance X = 4.38, X∑ = 1057 points; Lack of Control X = 3.37, X∑ = 814 points; Reduction in Other Activities X = 3.59, ∑ = 867 points; Time X = 3.80, X∑ = 917 points; Intention Effects X = 3.59, X∑ = 867 points. According to the arithmetic average of the 7 subclasses, only the Tolerance subclasses fall within the risk category (X ≥ 4) and in the non-dependent-symptomatic category (X ≤ 4), all subset members were included: Withdrawal Effects, Continuance dependence, Lack of Control, Reduction in Other Activities, Time and Intent Effects (Table 2). Despite the positive results highlighted by the 2 to 5 points, many students also awarded 1-point punches. The most striking incidence of 1 point reflects the mentality they practice to avoid feeling irritable, to avoid feeling anxious and when they are injured, to avoid pain and worsening health problems.

#### 3.1.2. The Compulsive Exercise Test (CET).

The overall assessment of CET (Table 2) revealed that the recorded values were statistically significant for *p* < 0.05, thus 3.34 ± 0.77; t (df 23) 21.18. The normality of the results for each item of the questionnaire highlighted by the Z values of the Kolmogorov-Smirnov test was statistically significant for *p* < 0.05. The Keiser-Meyer-Olkin measurements verified the sampling adequacy for analysis, KMO = 0.770, which were above Keiser’s criteria (>0.5). Bartlett’s test of sphericity χ2 (276) = 3369.813, *p* < 0.0001, indicated that correlations between items were sufficiently large for PCA. For the CET questionnaire, the Cronbach’s Alpha coefficient for 24 items was α = 0.868, suggesting that the questionnaire had a very high internal consistency. For subsections, the Cronbach’s Alpha coefficients were: Avoidance and rule-driven behavior α = 0.914, Weight control exercise α = 0.617, Mood improvement α = 0.758, Lack of exercise enjoyment α = 0.611, Exercise rigidity-dependent α = 0.675, suggesting that all the subscale of the questionnaire had high (over 0.600) and very high (over 0.800) internal consistency. The Cronbach’s Alpha coefficients were largely similar to the adult sample tested in the original development paper [26].

The percentage distribution of respondents for the entire CET questionnaire was as follows: 28.19% of subjects were awarded 5 points, 23.19% of subjects were awarded 4 points, 21.15% of subjects were awarded 3 points, 11.13% of subjects were awarded 2 points, 13.17% of subjects were awarded 1 point, and 1.83% of subjects were awarded 0 points. CET questionnaire (Table 2) revealed an average of X = 3.34 and an average sum of X∑ ± SD 808 ± 186.94 points. On the subscales of CET, the value of the recorded average was as follows: Avoidance and rule-driven behavior X = 3.08, XΣ = 745 points; Weight control exercise X = 3.21, X∑ = 775 points; Mood improvement X = 4.17, X∑ = 1006 points; Lack of exercise enjoyment X = 2.77, X∑ = 669 points; Exercise rigidity-dependent X = 3.49, X∑ = 841 points. According to the arithmetic average of the 5 subscales, only the Mood improvement subscale fell into the category of risk of exercise addiction (X ≥ 4) and in the category with potential risk (X ≤ 4) all the other subscales fell: avoidance and rule-driven behaviour, Weight control exercise, Exercise rigidity-dependent, and without risk (X ≤ 3) was only the subscale Lack of exercise enjoyment. The high percentage of 1 point given to items was in line with the mentality of the subjects by which if they missed a session of exercise, they would try and make up for it when they exercise, and if they cannot exercise, they feel agitated. The study also reflects the fact that 13.7% of the subjects did not enjoy exercising, perhaps considering this as a mandatory activity dedicated to improving health or physical condition and less to produce the enjoyment of exercise.

### 3.2. Body Perception

Analysis of the results (Table 3) on BMI points revealed that most subjects (36.1%) fell into the 23.7–25.8 category and the lowest number in the first category of ≤19.8 was only 1.7%. The most common silhouette identifying the subjects was silhouette number 5 with a percentage of 37.3% and the least selected by the subjects was silhouette number 1 in only the 1.7% cases. Most subjects, 38.6% of them, considered themselves satisfied with their body shape. In terms of motivation for weight control, most subjects believed they had the correct weight of 36.1% and only 8.7% thought they had to lose weight. An analysis between BMI scales and body self-image revealed that subjects with a BMI ≤ 19.8 identified themselves correctly with S1. BMI 19.9–21.1 category and S2 identified that 8 subjects overestimated their body image. Additionally, 7 subjects overestimated themselves; BMI 21.2–22.2 and S3, for BMI 22.2–23.6 and S4, 3 subjects undervalued themselves. For BMI 23.7–25.8 and S5, 3 subjects’ overestimated themselves, and 5 subjects undervalued themselves for BMI 25.9–28.1 and S6. For BMI ≥ 28.1 and S7, we identified 10 subjects who underestimated themselves. Of those who under-estimated themselves, most of the (10 (4.1%)) subjects had a BMI ≥ 28.1 and S7, and those who overestimate themselves were (8 (3.3%)) included in the BMI category of 19.9–21.1 and S2. In the assessment of the parameters evaluating the BMI, body self-imagine, perception of body shape, and weight motivation (Table 3), the recorded values were statistically significant for *p* < 0.05 and the normality of the results for each parameter highlighted by the Z values of the Kolmogorov–Smirnov test was statistically significant for *p* < 0.05, too. The BMI was 25.04 ± 3.36, which corresponded to silhouette number 5. Body Self-image was 4.50 ± 1.42, which corresponded to silhouette number 4. Perception of body shape recorded 3.66 ± 0.82 points, the rating being that of “satisfied”. With regard to the Weight motivation 3.00 ± 1.10, fitting into the “the weight is about right”.

We identified the following moderate correlations (between 0.5–0.7): between body self-image and BMI and a small negative correlation between BMI and perception of body shape (Table 4.). Low positive correlations (between 0.1–0.3) were between Body self-image and Perception of body shape as well as between BMI and Weight motivation. Very low correlations were recorded between Body self-image and Weight motivation, BMI and Weight motivation and Perception of body shape and Weight motivation. Statistically significant correlations were obtained only between Self-image body and BMI, Self-image body and Perception of body shape, as well as BMI and Perception of body shape. The sample of the study, according to the results of the study, had a correct perception of the body according to BMI and self-imagine.

### 3.3. Identification Preferences and Frequency of Use of the Fitness Monitoring Technology

The assessment of the Fitness Monitoring Technology questionnaire (Table 5.) revealed that the recorded values were statistically significant for *p* < 0.05, thus 2.46 ± 0.77; t (df 9) 19.53. The normality of the results for each item of the questionnaire highlighted by the Z values of the Kolmogorov–Smirnov test was statistically significant with *p* < 0.05. In the case of the group of students for the entire questionnaire, the Keiser-Meyer-Olkin measure, verified the sampling adequacy for analysis, KMO = 0.768, which were above the Keiser’s criteria (>0.5). Bartlett’s test of sphericity χ2 (45) = 1418.049, *p* < 0.0001, indicated that the correlation between items was sufficiently large for PCA. The Cronbach’s Alpha coefficient for 14 items of the fitness assessment questionnaire with monitoring was α = 0.831, suggesting that the questionnaire had a very high internal consistency. For subsections, the Cronbach’s Alpha coefficients were Fitness devices monitoring the performance on LCD monitors α = 0.765, and Fitness Devices with monitoring α = 0.856, suggesting that all subscales of the questionnaire had a high (over 0.600) and very high (over 0.800) internal consistency.

The analysis of the average percentage distribution of the subjects according to the score given by the Likert evaluation scale (1–5) on the entire questionnaire was the following: 9.37% of subjects were awarded 5 points, 15.02% of subjects were awarded 4 points, 27.67% of subjects were awarded 3 points, 14.64% of subjects were awarded 2 points, and 33.30% of subjects were awarded 1 point (Table 5). The assessment of the Fitness Monitoring Technology questionnaire (Table 5) recorded X = 2.46 points and an average of the sum of 592 ± 95.99 points. On the subscales, we obtained the values: for Fitness devices with performance monitoring on an LCD monitor, X = 2.68, X∑ = 647 points; and for Fitness devices with monitoring, X = 2.22, XΣ = 538 points, thus reflecting that the former was preferred by the students. Students participating in the study preferred to use predominantly bicycle treadmills and horizontal and elliptical bicycles, these being mainly recommended for improving cardiorespiratory capacity. The very low incidence of cardio tape use, the Heart Rate Monitor in the form of a bracelet or fitness watch and the Heart Rate Monitoring and Accelerometer reflected the fact that students have not formed a permanent exercise monitoring behaviour during training, despite their academic specialization.

Analysis of the correlation between the frequency of using fitness monitoring technologies and their types (Table 6) revealed a negative correlation for A5 exergames (*r* = −0.031). A small positive correlation was found for the following: D6 cardio, D7 fitness applications of Smartphone; D8 Heart Rate Monitoring, and Accelerometers. We found a medium correlation for A4 treadmills, D9 pedometers, and a large correlation for A1 treadmills and A3 elliptical bicycles. A very large correlation was found only for the A2 horizontal bicycle. All correlations were statistically significant, with only one exception for A5 where *p* = 0.627 (Table 6). The results reflect that study participants use fitness monitoring technologies with an insufficient frequency despite being aware of the benefits and importance of monitoring workouts during training.

### 3.4. Data Collection Instruments and Procedure

All data were processed online within a Google program which included the personal information (age, gender, weight, height, and institutional membership), Exercise Dependence Scale-R (EDS-21), Compulsive Exercise Test (CET)*,* Body perceptions and Fitness Monitoring Technology. The semantic and technical equivalence of the EDS-21 and CET questionnaires were respected, the questionnaires have been translated and verified in Romanian by two authorized translators (native Romanian) and English language university teachers from the English department.

#### 3.4.1. Exercise Dependence Attitudes

For exercise dependence attitudes we used two questionnaires: the Exercise Dependence Scale-R (EDS-21) and Compulsive Exercise Test (CET). We decided to use two questionnaires to highlight the exercise addiction attitude because they target different psychometric properties. The Exercise Dependence Scale-R (EDS-21) [38]. The EDS-R consists of 21 items scored on a 6-point Likert scale, ranging from 1 (never) to 6 (always). The questionnaire was organized in 7 subscales: Withdrawal Effects; Continuous dependence; Tolerance highlighted; Lack of Control according; Reduction in Other Activities; the Time given and Intention Effects. Classifying criteria for some behavioral attitudes were as follows: the dependent range is operationalized as indicating a score of 5 or 6 for that item. Individuals who scored 3 to 4 range were classified as symptomatic. These individuals may theoretically be considered at risk for exercise dependence. Finally, individuals who scored in the 1–2 range were classified as asymptomatic [66]. EDS-21 targets: overall score of exercise dependence symptoms; differentiates between at-risk for exercise dependence, nondependent-symptomatic, and nondependent-asymptomatic; specifies whether individuals had evidence of physiological dependence (i.e., evidence of tolerance or withdrawal) or no physiological dependence (i.e., no evidence of tolerance or withdrawal) [67,68].

The Compulsive Exercise Test (CET) targeted participants’ levels of compulsive exercise [28]. The CET consisted of 24 items scored on the Likert scale using a 6-point scale, ranging from 0 (never true) to 5 (always true). CET was structured on the following 5 subscales: avoidance and rule-driven behavior; weight control exercise; mood improvement; lack of exercise enjoyment and exercise rigidity depending [29]. High scores indicated high levels of compulsive exercise. Subjects were classified in three categories: risk-free, risk-bearing and the addicted ones were made according to the average of the points recorded on the whole questionnaire as follows: average of ≤3 points for the risk-free category, 3–5 points in the risk category and over 5 points in the compulsive exercise category.

#### 3.4.2. Body Perception

The body shape assessment included the following: BMI, identifying the corresponding self-image of body image, analyzing the answers regarding satisfaction degree of body shape, and body weight motivation. To evaluate BMI and body image silhouettes, we took into consideration 7 criteria of appreciation: 1 silhouette (S1) corresponds to a BMI ≤ 19.8; 2 silhouettes (S2) correspond to BMI 19.9–21.1; 3 silhouettes (S3) correspond to BMI 21.2–22.2; 4 silhouettes (S4) correspond to 22.2–23.6; 5 silhouettes (S5) correspond to BMI 23.7–25.8; 6 silhouettes (S6) correspond to BMI 25.9–28.1, and 7 silhouettes (S7) in BMI correspond to ≥28.2. The Perception on body shape consisted of the Likert scale using a 5-point scale, ranging from (1 point) would like to be more slim; (2 points) I would like to be a little bit slim, (3 points) My weight is about right; (4 points) I would like to be a little big; (5 points) I would like to be more big. The Weight motivation consisted in one item scored with 5 points on the Likert scale ranging from (1 point) dissatisfied with the way I look; (2 points) relatively dissatisfied; (3 points) satisfied; (4 points) content; (5 points) very satisfied of the way I look.

#### 3.4.3. The Fitness Monitoring Technology

The Fitness Monitoring Technology questionnaire aimed to identify preferences and frequencies of fitness monitoring technology usage. The items of the questionnaire and the evaluation scale were designed in collaboration with the specialists from the department of psycho-pedagogy (the specialist poses the competence to design and validate the evaluation tools) and with those from the department of physical education and sport at the University of Medicine, Pharmacy, Sciences and Technology of Targu Mures. The elaboration of the questionnaire was made in accordance with specialized literature and aimed at identifying the most used and trending fitness monitoring technologies used in the fitness centers. The questionnaire included 11 items scoring 5 points on the Likert scale, ranging from 1 (never) to 5 (always). The questionnaire for evaluating the preferences and frequency of use of fitness with monitoring technology is structured on the following three subscales: fitness equipment with monitoring items 1–5, fitness devices with monitoring items 6–10, frequency of use of fitness equipment for item 11.

### 3.5. Statistical Analyses

The results of the study were statistically processed using IBM SPSS Statistics 22. The statistical analysis included the following index: the arithmetic average (X), standard deviation (SD), t-Student test (*t*), sum (∑) and Kolmogorov-Smirnov Test (Z), the significance threshold was *p* < 0.05. The Pearson index (r) was used to reveal the correlation between parameters of the study. We calculated the PCA for the data set: the Bartlett’s sphericity test and the Kaiser-Meyer-Olkin (KMO). The reliability or the internal consistency of the questionnaire was calculated using the Cronbach’s alpha statistical index (α). To evaluate the relevance of the items, we calculated the average and sum of points and the percentage assigned by the subjects of the study.

## 4. Discussion

### 4.1. Exercise Dependence

We considered that the high incidence of our students were extrinsic motivations based on the lack of mentalities according to the way of putting the scientific knowledge of physical education and health which they learned during their academic studies into practice. It is worth noting that the values recorded in our study were much higher than those of physical education and sports students and other specialties or among young people who regularly practiced recreational activities. In this regard, following the analysis of the EDS-21 results, we identified that 48 subjects (20%) were in the non-dependent and asymptomatic category, 172 (71.7%) of the students fell into the non-dependent and symptomatic category, and 21 subjects (8.7%) were in the at-risk for exercise dependence category. About the CET, the results show that 50 subjects (20%) were in the category of risk-free dependence attitudes, 159 (66%) of students fell into the category of risk of exercise dependence, and 32 subjects (13.3%) were identified as already dependent on exercise. In support of our findings, Szabo, A et al. [3], found that an average score of 6.9% of students was addicted to physical exercise; Hausenblas and Symons Downs [8], revealed that 2.5% of their university student sample could be classified as exercise dependent; Terry et al. found that 3.0% of the university students were at risk of exercise addiction [69].

Regarding the incidence of students identified with exercise addiction in our study, we believe that they fell within the identified values in previous studies on fitness instructors, personal trainers, and athlete performers. Very close to our results of prevalence of exercise dependency, but lower than ours, were identified by Lichtenstein et al. [33] who investigated 577 subjects and found that 6% of those that practiced low-intensity activities and 7% of those that practiced high-intensity activities were at risk of exercise addiction. The results of our study are much closer to those of the fitness practitioners identified by Downs D.S. et al. [38], who found that the prevalence of exercise dependency was 7.1% in football and 9.7% in fitness. Some results of our study are not in line with previous studies which focused on youngsters who were recruited from fitness centers and who practiced recreational sports, thus Szabo A. at al. [70] found between 1.9–3.2% subjects were exercise addicted, and Costa S. et al. stated that 4.4% of subjects were exercise addicted [35]. The values recorded in our study were much higher than those of students from other specialties, or among young people who practice recreational physical activity on a regular basis. Regular physical exercise has a major influence on physical and mental health optimization [71,72]. Intrinsic and extrinsic motivation, and the pursuit of immediate and powerful satisfaction, turn more and more practitioners into physical exercise addicts [73]. Recent studies have signaled an alarm on the emergence of a problematic reality for the health of today’s and future society [74].

### 4.2. Body Perception

The results of our study reflected a good self-image of body shape perception and our results are in the line with the results of previous studies regarding students of physical education, regular physical activity practitioners, and young athletes. In our study, we identified statistically significant correlations between the Self-image body and BMI, Body self-image and Perception of body shape, as well as BMI and Perception of body shape. Regarding the correct association between BMI and body image (silhouette), we identified that 18 (7.4%) of subjects overestimated themselves and 18 (7.4%) of subjects underestimated themselves. Most subjects considered themselves satisfied with their body shape (38.6%), 17% were very satisfied, and none were dissatisfied with their physical appearance. In terms of motivation for weight control, most subjects believed they had the correct weight of 36.1% and only 8.7% thought they had to lose weight. Our study is in line with other studies developed in European countries. In this regard, a study of students from Denmark (548) and England (816) found that perceptions of body image varied as follows: 8.6% were too thin, 37.7% were “just right”, and 53.7% were “too fat” [50]. Another study in line with our results reported 45% of participants were assessed exactly as they perceived themselves, without any gender differences, the rest of the people thought they were seen as being fat [40,75]. In agreement with our study, several other studies have shown that non-practicing students tended to have a higher fat body perception compared to those who practiced sports and who chose normal, regular, athletic patterns [76,77,78]. Our students of physical education had an almost correct body image, but our results were not in accordance with studies of students of others specialization who were much more disappointed with their body image [79,80].

### 4.3. Using Fitness Technology with Monitoring

The results reflect that the study participants used insufficient fitness monitoring technologies despite their academic training. This part of the study aimed at identifying the level of use and preference for the use of different physical training monitoring devices. The results of the study revealed that only 11.6% of students used fitness monitoring technology and 17% never used these technologies during physical training. Regarding the preference of using fitness monitoring technologies, we identified that the most preferred monitor devices were treadmills, horizontal bicycles, and elliptical bicycle; the favorite monitor devices were: Smartphone fitness applications, pedometer and Heart Rate Monitoring, and Accelerometers. The results were surprising in terms of the percentage of subjects who never used such technology. We also identified a lower percentage of students in the study group using fitness monitoring technology compared to evidence identified by other studies [81,82,83,84]. Our results were not in line with previous studies which found that 71% of the study participants appreciated the use of smartphone apps for weight loss, increased physical activity index, improved dietary habits, reduced body mass index, etc. [83]; 19.6% used some type of fitness device; 13.8% used a calorie tracking device [84]; 38% used exercise/fitness apps, 31% used nutrition/calorie counter apps, and 12% used weight loss apps [85], and few studies recommend it to improve the physical capacity and health [86,87,88]. Despite the specialty knowledge of our students, the low level of using technologies may have a justification in some misconceptions about the efficiency and validity of these technologies, maybe based on recent studies [54,89,90].

### 4.4. Limits and Strengths

Limitations. It would be useful to expand the study by involving a larger number of students from physical education and sports specializations, as well as from other specializations. The evaluation via questionnaires of exercise dependence attitudes is limited because those who fell into this category in a major percentage they practiced individually and it was very difficult to include them in a study. In our study, we wanted to highlight the frequency and preference for using fitness technology with monitoring, but future studies could focus on extending the assessment of the accuracy of the monitoring according to the parameters of the effort and the impact of their use in terms of health.

Strengths: a major strength is the complexity of the analyzed information regarding the incidence of exercise dependence on students of physical education and sports. The results of body perception allowed us to identify the level of body image appreciation, body weight, and body shape perception. Highlighting the preference and frequency in using fitness monitoring technology allowed us to identify the impact technology has on sports training and health monitoring.

## 5. Conclusions

Our study has identified the implications of exercise addiction, the level of body perception, and the use of fitness monitoring technologies by physical education and sports students who should be able to control their behavior in exercising, to have a correct body perception, and to be able to use modern technology correctly and frequently in order to diminish the medical incidence of compulsive behavior dependence. Following the analysis of the EDS-21, only 21 subjects (8.7%) were at-risk for exercise addiction and CET 32 subjects (13.3%) are identified as already dependent on exercise. The results are over our expectation because the knowledge and competence of our students as future specialists of physical education requires promoting active and correct behaviors and lifestyles. The high incidence of identified subjects with the incidence of compulsive behavioral dependence may have multiple intrinsic and extrinsic causes due to the current trend in fitness by exacerbating cult for physical fitness and for a more defined and muscular body shape.

Physical and sport education students have a fairly accurate body perception, they have a self-image close to the real one and most of them were satisfied with their body shape. The evaluation of preferences and frequencies in the use of fitness technology with monitoring highlight the diversity of subjects’ attitudes, revealing that a relatively large percentage of subjects did not use these technologies at all. Students from the study, despite their academic expertise, are not sufficiently aware of the need to monitor the body’s functional parameters during exercise. The use of modern technologies contributed to the real-time monitoring of the body’s functional parameters during exercise, facilitating the adjustment of the intensity, volume, and complexity of physical programs to the level of training, age specificities, and expected performance goals. We recommend the need to extend and promote programs such as health education counseling, psychological counseling, nutrition counseling, and sports counseling. All these programs aim at reducing the risk and incidence of exercise addiction, promote healthy physical programs according to the World Health Organization recommendations, and focus subjects on developing self-esteem without concentrating on some aspects of body image.

## Figures and Tables

**Table 1:** The statistical analysis of the average responses according to the Likert scale (6) per item in the questionnaire evaluating the exercise dependence attitudes (EDS-21).

Sub-Scale	Items	X ± SD	∑	*t*	*p*	Likert Scale Points					
						6 Points *N* (%)	5 Points *N* (%)	4 Points *N* (%)	3 Points *N* (%)	2 Points *N (%)*	1 Point *N (%)*
WE	1. I exercise to avoid feeling irritable.	3.10 ± 1.58	749	30.48	0.00	13 (5.4)	41 (17)	**56 (23.2)**	38 (15.8)	35 (14.5)	**58 (24.1)**
	8. I exercise to avoid feeling anxious.	2.75 ± 1.60	665	26.61	0.00	22 (4.6)	28 (11.6)	54 (22.4)	32 (13.3)	31 (12.9)	**85 (35.3)**
	15. I exercise to avoid feeling tense.	3.45 ± 1.58	833	33.77	0.00	30 (12.4)	42 (17.4)	50 (20.7)	32 (13.3)	60 (24.9)	27 (11.2)
CD	2. I exercise despite recurring physical problems.	3.63 ± 1.60	876	35.23	0.00	30 (12.4)	56 (23.2)	52 (21.6)	35 (14.5)	35 (14.5)	33 (13.7)
	9. I exercise when injured	3.37 ± 1.78	814	29.30	0.00	35 (14.5)	48 (19.9)	39 (16.2)	24 (10)	41 (17)	**54 (22.4)**
	16. I exercise despite persistent physical problems.	3.34 ± 1.70	805	30.49	0.00	24 (10)	**60 (24.9)**	31 (12.9)	32 (13.3)	47 (19.5)	47 (19.5)
T	3. I continually increase my exercise intensity to achieve the desired effects/benefits.	4.73 ± 1.16	1141	63.16	0.00	**82 (34)**	**62 (25.7)**	**56 (23.2)**	33 (13.7)	8 (3.3)	-
	10. I continually increase my exercise frequency to achieve the desired effects/benefits	4.34 ± 1.44	1047	46.61	0.00	**70 (29)**	59 (24.5)	30 (12.4)	52 (21.6)	26 (10.8)	4 (1.7)
	17. continually increase my exercise duration to achieve the desired effects/benefits.	4.08 ± 1.41	985	44.90	0.00	44 (18.3)	**62 (25.7)**	54 (22.4)	42 (17.4)	30 (12.4)	9 (3.7)
LC	4. I am unable to reduce how long I exercise.	3.67 ± 1.21	885	46.73	0.00	19 (7.9)	39 (16.2)	**75 (31.1)**	**68 (28.2)**	32 (13.3)	8 (3.3)
	11. I am unable to reduce how often I exercise.	3.40 ± 1.36	821	38.79	0.00	24 (10)	26 (10.8)	53 (22)	**76 (31.5)**	45 (18.7)	17 (7.1)
	18. I am unable to reduce how intense I exercise.	3.05 ± 1.30	737	36.27	0.00	8 (3.3)	31 (12.9)	48 (19.9)	58 (24.1)	**72 (29.9)**	24 (10)
ROA	5. I would rather exercise than spend time with family/friends.	3.20 ± 1.26	772	39.26	0.00	12 (5)	24 (10)	54 (22.4)	**86 (35.7)**	41 (17)	24 (10)
	12. I think about exercise when I should be concentrating on school/work.	3.49 ± 1.60	842	33.74	0.00	32 (13.3)	35 (14.5)	**66 (27.4)**	31 (12.9)	41 (17)	36 (14.9)
	19. I choose to exercise so that I can get out of spending time with family/friends	4.09 ± 1.67	988	38.10	0.00	**72 (29.9)**	41 (17)	34 (14.1)	51 (21.2)	19 (7.9)	24 (10)
TI	6. I spend a lot of time exercising.	4.11 ± 1.33	992	47.92	0.00	49 (20.3)	50 (20.7)	51 (21.2)	65 (27)	23 (9.5)	3 (1.2)
	13. I spend most of my free time exercising.	3.66 ± 1.53	883	37.05	0.00	32 (13.3)	49 (20.3)	**56 (23.2)**	35 (14.5)	48 (19.9)	21 (8.7)
	20. A great deal of my time is spent exercising.	3.63 ± 1.34	876	41.87	0.00	24 (10)	46 (19.1)	52 (21.6)	65 (27)	45 (18.7)	9 (3.7)
IE	7. I exercise longer than I intend.	3.43 ± 1.52	828	35.00	0.00	35 (14.5)	32 (13.3)	34 (14.1)	56 (23.2)	**70 (29)**	14 (5.8)
	14. I exercise longer than I plan.	3.95 ± 1.51	953	40.53	0.00	53 (22)	39 (16.2)	54 (22.4)	45 (18.7)	39 (16.2)	11 (4.6)
	21. I exercise longer than I expect.	3.40 ± 1.40	821	37.70	0.00	23 (9.5)	35 (14.5)	49 (20.3)	57 (23.7)	**64 (26.6)**	13 (5.4)

X = mean of points, SD = standard deviation, ∑ = sum of points, *p* = probability level, *t* = Student test values, N = number of subjects, % = percentage of total responses per item, with bold I highlighted the best 3 results reported to Likert scale (1–6). WE = Withdrawal Effects; CD = Continuance dependence, T = Tolerance; LC = Lack of Control; ROA = Reduction in Other Activities, TI = Time, IE = Intention Effects.

**Table 2:** The statistical analysis of the average responses according to the Likert scale (6) per item in the questionnaire evaluating the Compulsive Exercise Test (CET).

Sub-Scale	Items	X ± SD	∑	*t*	*p*	Likert Scale Points					
						5 Points *N (%)*	4 Points *N (%)*	3 Points *N (%)*	2 Points *N (%)*	1 Point *N (%)*	0 Point *N (%)*
ARDB	9. If I cannot exercise, I feel low or depressed	3.11 ± 1.36	751	35.45	0.00	40 (16.6)	**80 (33.2**)	28 (11.6)	**54 (22.4**)	39 (16.2)	-
	10. I feel extremely guilty if I miss an exercise session	3.16 ± 1.42	763	34.56	0.00	44 (18.3)	78 (32.4)	45 (18.7)	26 (10.8)	44 (18.3)	4 (1.7)
	11. I usually continue to exercise despite injury or illness, unless I am very ill or too injured	3.24 ± 1.28	781	39.06	0.00	44 (18.3)	69 (28.6)	58 (24.5)	43 (17.8)	22 (9.1)	4 (1.7)
	15. If I miss an exercise session, I will try and make up for it when I next exercise	3.32 ± 1.39	802	37.03	0.00	50 (20.7)	**87 (36.1)**	37 (16.1)	34 (14.1)	25 (10.4)	**8 (3.3)**
	16. If I cannot exercise, I feel agitated	2.90 ± 1.41	699	31.78	0.00	38 (15.8)	52 (21.6)	55 (22.8)	48 (19.9)	40 (16.6)	**8 (3.3)**
	20. If I cannot exercise, I feel angry and/or frustrated	3.00 ± 1.40	723	33.07	0.00	46 (19.1)	46 (19.1)	59 (24.5)	48 (19.9)	36 (14.9)	6 (2.5)
	22. I feel like I’ve let myself down if I miss an exercise session	2.90 ± 1.34	700	33.57	0.00	27 (11.2)	63 (26.1)	66 (27.4)	37 (15.4)	41 (17)	7 (2.9)
	23. If I cannot exercise, I feel anxious	3.08 ± 1.35	743	35.25	0.00	48 (19.9)	46 (19.1)	62 (25.7)	**53 (22)**	27 (11.2)	5 (2.1)
WCE	2. I exercise to improve my appearance	4.31 ± .93	1039	71.26	0.00	132 (54.8)	71 (29.5)	23 (9.5)	11 (4.6)	4 (1.7)	-
	6. I feel I have eaten too much, I will do more exercise	2.80 ± 1.33	677	32.68	0.00	30 (12.4)	44 (18.3)	77 (32)	13 (12.9)	**58 (24.1)**	1 (0.4)
	8. I do not exercise to be slim	2.77 ± 1.16	668	37.00	0.00	16 (6.6)	51 (21.2)	**78 (32.4)**	**54 (22.4)**	42 (17.4)	-
	13. I exercise to burn calories and lose weight	3.38 ± 1.32	815	39.64	0.00	42 (17.4)	**99 (41.1**)	52 (21.6)	11 (4.6)	31 (12.9)	6 (2.5)
	18. if I cannot exercise, I worry that I will gain weight	2.81 ± 1.46	678	29.88	0.00	30 (12.4)	68 (28.2)	41 (17)	40 (16.6)	53 (22)	9 (3.7)
MI	1. I feel happier and/or more positive after I exercise	4.61 ± 0.64	1112	110.35	0.00	**170 (70.5)**	49 (20.3)	22 (9.1)	-	-	-
	4. I feel less anxious after I exercise	3.77 ± 1.24	910	47.09	0.00	92 (38.2)	59 (24.5)	49 (20.3)	28 (11.6)	11 (4.6)	2 (0.8)
	14. I feel less stressed and/or tense after I exercise	4.31 ± 0.96	1039	69.33	0.00	140 (58.1)	50 (20.7)	44 (18.3)	-	7 (2.9)	-
	17. Exercise improves my mood	4.53 ± 0.68	1092	102.05	0.00	**155 (64.3)**	59 (24.5)	27 (11.3)	-	-	-
	24. I feel less depressed or low after I exercise	3.65 ± 1.37	880	41.09	0.00	88 (36.5)	59 (24.5)	48 (19.9)	17 (7.1)	26 (10.8)	3 (1.2)
LEE	5. I find exercise a chore	2.19 ± 1.31	530	26.04	0.00	7 (2.9)	48 (19.9)	42 (17.4)	40 (16.6)	**97 (40.2)**	7 (2.9)
	12. I do not enjoy exercising	4.66 ± 0.66	1125	108.30	0.00	**184 (76.3**)	38 (15.8)	15 (6.2)	4 (1.7)	-	-
	21. I enjoy exercising	1.46 ± 1.11	352	20.35	0.00	4 (1.7)	10 (4.1)	32 (13.3)	34 (14.1)	**128 (53.1**)	**33 (13.7)**
ERD	3. I like my days to be organized and structured of which exercise is just one part	4.01 ± 1.02	968	61.10	0.00	104 (43.2)	60 (24.9)	54 (22.4)	23 (9.5)	-	-
	7. My weekly pattern of exercise is repetitive	3.30 ± 1.10	796	46.57	0.00	31 (12.9)	78 (32.4)	**84 (34.9)**	32 (13.3)	13 (5.4)	3 (1.2)
	19. I follow a set routine for my exercise sessions, e.g., walk or run the same route e.g., walk or run the same route etc.	3.15 ± 1.17	761	41.67	0.00	34 (14.1)	51 (21.2)	**103 (42.7)**	32 (13.3)	14 (5.8)	7 (2.9)

X = average of points, SD = standard deviation, ∑ = sum of points, *p* = probability level, *t* = Student test values, N = number of subjects, % = percentage of total responses per item, with bold I highlight the best 3 results reported to Likert scale (0–5). ARDB = Avoidance and rule-driven behavior; WCE = Weight control exercise, MI = Mood improvement; LEE = Lack of exercise enjoyment; ERD = Exercise rigidity-dependent.

**Table 3:** The statistical analysis of data according to the evaluation of the body perception criteria.

Items	Response Weighting								X ± SD	*t*	*p*
BMI	Criteria	≤19.8	19.9–21.1	21.2–22.2	22.2–23.6	23.7–25.8	25.9–28.1	≥28.1	25.04 ± 3.36	115.49	0.00
	*N (%)*	4 (1.7)	19 (7.9)	16 (6,6)	48 (19.9)	87 (36.1)	40 (16.6)	27 (11.2)			
Body self-imagine (silhouette)	Criteria	S1	S2	S3	S4	S5	S6	S7	4.50 ± 1.42	49.04	0.00
	*N (%)*	4 (1.7)	27 (11.2)	23 (9.5)	45 (18.7)	90 (37.3)	35 (14.5)	17 (7.1)			
Perception of body shape	Criteria	5 point	4 points		3 points	2 points	1 point		3.66 ± 0.82	69.00	0.00
	*N (%)*	41 (17)	93 (38.6)		93 (38.6)	14 (5.8)	-				
Weight motivation	Criteria	5 point	4 points		3 points	2 points	1 point		3.00 ± 1.10	42.06	0.00
	*N (%)*	26 (10.8)	49 (20.3)		87 (36.1)	58 (24.1)	21 (8.7)				

N = Number of subject, % = percentage, S = silhouette number, BMI = body mass index, X = mean, SD = standard deviation, *p* = probability level, *t* = one simple Student *t*-test values.

**Table 4:** The statistical correlation between BMI, body self-imagine, perception of body shape, and weight motivation.

Parameters	BMI	Perception of Body Shape	Weight Motivation
Body self-imagine	0.753 **	−0.200 **	0.014
BMI	-	−0.225 **	0.077
Perception of body shape	-	-	−0.008

** Pearson Correlation is significant at the 0.01 level (2-tailed), BMI = body mass index.

**Table 5:** The statistical analysis of the average responses according to the Likert scale (5) per item in the assessment of the use of fitness technology with the monitoring questionnaire.

Subscale	Items	X ± SD	∑	*t*	*p*	Likert Scale Points				
						5 Points *N (%)*	4 Points *N (%)*	3 Points *N (%)*	2 Points *N (%)*	1 Point *N (%)*
FD-LCD	1. Do you use treadmills while exercising?	3.00 ± 1.34	725.00	34.81	0.00	**42 (17.4)**	51 (21.2)	55 (22.8)	**53 (22)**	40 (16.6)
	2. Do you use the horizontal bike while practicing?	2.89 ± 1.29	697.00	34.72	0.00	**28 (11.6)**	**60 (24.9)**	56 (23.2)	52 (21.6)	45 (18.7)
	3. Do you use elliptical bicycle during exercise?	2.55 ± 1.35	616.00	29.25	0.00	**28 (11.6)**	31 (12.9)	64 (26.6)	42 (17.4)	76 (31.5)
	4. Do you use while training the (paddle machine)?	2.19 ± 1.13	528.00	29.86	0.00	12 (5)	12 (5)	**75 (31.1)**	**53 (22)**	89 (36.9)
	5. Do you use exergames during exercise?	2.78 ± 1.19	672.00	36.35	0.00	20 (8.3)	38 (15.8)	**106 (44)**	25 (10.4)	52 (21.6)
FDM	6. Do you use cardio tape during exercise?	1.86 ± 1.04	450	27.65	0.00	8 (3.3)	-	70 (29)	37 (15.4)	**126 (52.3)**
	7. Do you use the fitness apps of your Smartphone while practicing?	2.68 ± 1.33	646	31.21	0.00	19 (7.9)	**56 (23.2)**	69 (28.6)	23 (9.5)	74 (30.7)
	8. Do you use the Heart Rate Monitor in the form of a bracelet or a fitness watch during exercise?	2.10 ± 1.21	508	26.898	0.00	8 (3.3)	24 (10)	**72 (29.9)**	19 (7.9)	**118 (49)**
	9. Do you use pedometers during exercise?	2.52 ± 1.33	608	29.323	0.00	27 (11.2)	35 (14.5)	43 (17.8)	**68 (28.2)**	68 (28.2)
	10. Do you use the Heart Rate Monitoring and Accelerometer during exercise?	1.98 ± 1.32	479	23.242	0.00	22 (9.1)	11 (4.6)	45 (18.7)	27 (11.2)	**136 (56.2)**
FU	How often do you use machines or devices in training?	2.91 ± 1.27	702	35.51	0.00	**28 (11.6)**	**60 (24.9)**	57 (23.7)	**55 (22.8)**	41 (17)

A, D = code of items, X = average of points, SD = standard deviation, ∑ = sum of points, *p* = probability level, *t* = Student test values, N = number of subjects, % = percentage of total responses per item, with bold I highlighted the best 3 results reported to the Likert scale (1–5). FD-LCD = Fitness devices with LCD monitor performance; FDM = Fitness monitoring devices, FU = Frequency of using fitness equipment.

**Table 6:** The Pearson correlations of the frequency of use of fitness monitoring technology and their typology (items A = devices, D = devices).

Items	A1	A2	A3	A4	A5	D6	D7	D8	D9	D10
Frequency	0.744 **	0.991 **	0.771 **	0.489 **	−0.031	0.129 *	0.158 *	0.184 **	0.353 **	0.169 **

A, D = code of items, ** Correlation is significant at the 0.01 level (2-tailed), * Correlation is significant at the 0.05 level (2-tailed).
